# 292 Activating community health workers: A community-academic partnership to understand vaccine hesitancy – CORRIGENDUM

**DOI:** 10.1017/cts.2024.538

**Published:** 2024-05-13

**Authors:** Caesar Thompson, Emily Stiehl, Mark Dworkin, Nadine Peacock, Naseem Parsa, Melissa Martin, Cornelius Chandler, Diana Ghebenei, Jennifer Hebert-Beirne

The above abstract published with an error in the author list. Devyani Gore was incorrectly shown as the first author. The first author is Caesar Thompson.

The original abstract has been corrected online to rectify this error.
